# Comparing the mechanical energetics of walking among individuals with unilateral transfemoral limb loss using socket and osseointegrated prosthetic interfaces

**DOI:** 10.1038/s41598-025-93211-1

**Published:** 2025-03-21

**Authors:** Pawel R. Golyski, Benjamin K. Potter, Jonathan A. Forsberg, Brad D. Hendershot

**Affiliations:** 1 Extremity Trauma and Amputation Center of Excellence, Falls Church, VA USA; 2 Walter Reed National Military Medical Center, Bethesda, MD USA; 3 Uniformed Services University of Health Sciences, Bethesda, MD USA; 4 University of Pennsylvania, Philadelphia, PA USA; 5 Orthopaedics, Department of Surgery, Memorial Sloan Kettering Cancer Center, New York, NY USA

**Keywords:** Bone-anchored prostheses, Mechanical work, Ground reaction forces, Lower limb amputation, Biomechanics, Biomedical engineering, Mechanical engineering, Musculoskeletal models

## Abstract

Osseointegration (OI), or bone-anchoring, of a prosthesis is a transformative procedure for addressing issues of socket fit among individuals with lower limb loss. Mechanically, the removal of the socket interface substantially alters the transmission of load and the flow of energy through the prosthetic limb. Here, we compared the mechanical energetics of walking between socket and OI interfaces using biomechanical data and custom models of 8 service members pre- and ~24-months post-OI. Relative to a socket interface, an OI interface shifted loads toward the intact limb, which increased collision losses, while the net mechanical work of both the prosthetic and intact limbs remained minimal for both interfaces. At the joint level, despite the removal of the socket interface potentially saving ~ 6 J of work per stride, these reduced collision losses were transmitted to the center of mass instead of altering joint work. The principal change in prosthetic limb joint mechanics with an OI interface was a decrease in negative prosthetic limb hip work during late stance, driven by a decreased hip flexion moment and prosthetic limb offloading. Our findings suggest that despite previously reported improvements in walking economy, after OI individuals likely walk with increased mechanical energetic asymmetry.

## Introduction

Osseointegration (OI), or bone-anchoring, of a lower limb prosthesis can provide a valuable alternative to standard socket-based suspension to address issues of poor socket fit and residual limb health^1^. For individuals with transfemoral limb loss, OI has demonstrated improvements in an array of outcomes, including increased hip range of motion^2^, quality of life^3–6^, mobility^7–9^, and prosthesis embodiment^10^. Biomechanically, direct fixation of a transfemoral prosthesis removes six degrees of freedom between the socket and the residual femur, which can drastically alter the transmission and sensation of force between the environment and person^11,12^. Removing these degrees of freedom also has an important implication on the flow of mechanical energy through the prosthetic limb, as it also removes an interface that can dissipate energy by compressing under high loads (i.e., bodyweight in early stance) and extending under low loads (i.e., prosthesis mass during swing)^13^.

Although the negative work at a transfemoral socket interface has not been directly quantified in literature, it can be estimated by combining the proximal displacement of the socket relative to the femur (1.6 cm over 20% of the gait cycle)^13,14^, the vertical ground reaction force at the time of peak displacement (90% BW, or 769 N at 20% of the gait cycle)^11^, and the duration of an average gait cycle (1.36 s)^15^, during self-selected walking in this population. Assuming cubic profiles for displacement and load, with constraints of zero velocity and load rate at both initial contact and 20% of the gait cycle, ~ 6 J of negative work per stride can be attributed to the socket interface, which would equate to ~ 45% of negative collision work during walking in individuals without limb loss^16^. Given that negative collision work is a determinant of the metabolic cost of walking^17^, energetic losses at the socket interface may incur appreciable metabolic penalties^18^.

During steady-state locomotion, the limbs are sensitive to sources and sinks of mechanical energy since the energetic objective at the whole-body level is strictly defined – during walking at a constant speed on level ground, the net mechanical work performed on the whole-body center of mass (COM) by external forces (i.e., both limb GRFs in unassisted walking) is zero, on average over several strides. For healthy, uninjured individuals, the limbs behave symmetrically and contribute to COM work equally, and thus each limb’s net work on the COM over a stride is also zero on average. For individuals with unilateral lower limb loss, symmetry is disrupted through reduced prosthetic foot push-off work, which increases collision losses on the intact limb relative to uninjured levels^19,20^. Despite this disruption, individuals with unilateral transtibial limb loss have also demonstrated reduced collision losses on the prosthetic limb, such that positive midstance and push-off work approximately offset negative collision work within each limb^19,21^. Equivalent contributions by each limb need not be the case, however, as demonstrated by the net positive and negative work contributions over a stride for the non-paretic and paretic limbs of stroke survivors^22,23^. An additional consideration relating to overall limb mechanical energetics for individuals with unilateral transfemoral limb loss is a tendency to preferentially load the intact limb relative to the prosthetic limb post- vs. pre-OI^11^, with exceptions^24^. Off-loading of the prosthetic limb should decrease collision losses and associated joint demands on the prosthetic side but increase such demands on the intact side. Further, among individuals using conventional, energetically passive prosthetic components (e.g., mechanical or microprocessor knees and energy storing and returning feet), if similar net prosthetic limb work is maintained pre- to post-OI, removal of an energetically “lossy” socket interface should also reduce the positive work demands on the prosthetic-side hip joint.

**Fig. 1:**
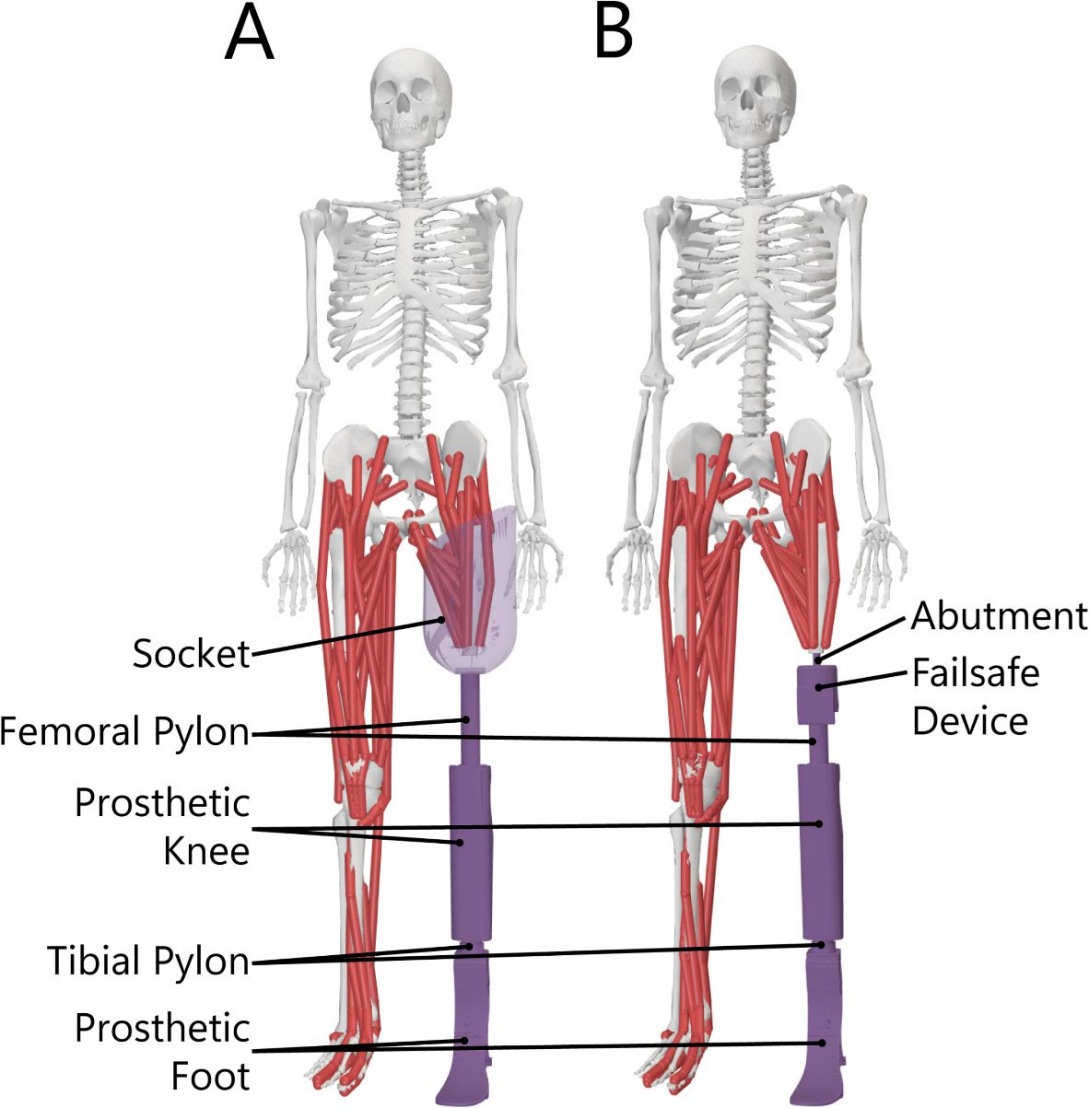
Custom musculoskeletal models of individuals with unilateral transfemoral amputation (**A**) using socket suspension, with a translational degree of freedom between the socket and residual femur directed along the long axis of the femur, and (**B**) with an osseointegrated interface, where the residual femur is rigidly attached to the abutment and prosthesis. This figure was generated using OpenSim 4.4^25^ (https://simtk.org/projects/opensim) and Adobe Illustrator 2024 (https://www.adobe.com/products/illustrator.html).

Given the above rationale, three hypotheses were addressed using custom full-body models of individuals with unilateral transfemoral limb loss using socket and OI interfaces (Fig. 1): (H1) net work over a stride of the prosthetic and intact limbs would be similar, and therefore approximately zero, for both interfaces; (H2) a shift toward greater intact limb loading with an OI interface would elicit larger positive and negative work demands on the intact limb and smaller demands on the prosthetic limb; (H3) there would be decreased positive work demands at the hip of the prosthetic limb with an OI interface.

## Results

### With both socket and osseointegrated interfaces, net limb work is similar and minimal, but positive and negative components are not, due to a shift toward greater intact limb loads post-osseointegration

**Fig. 2:**
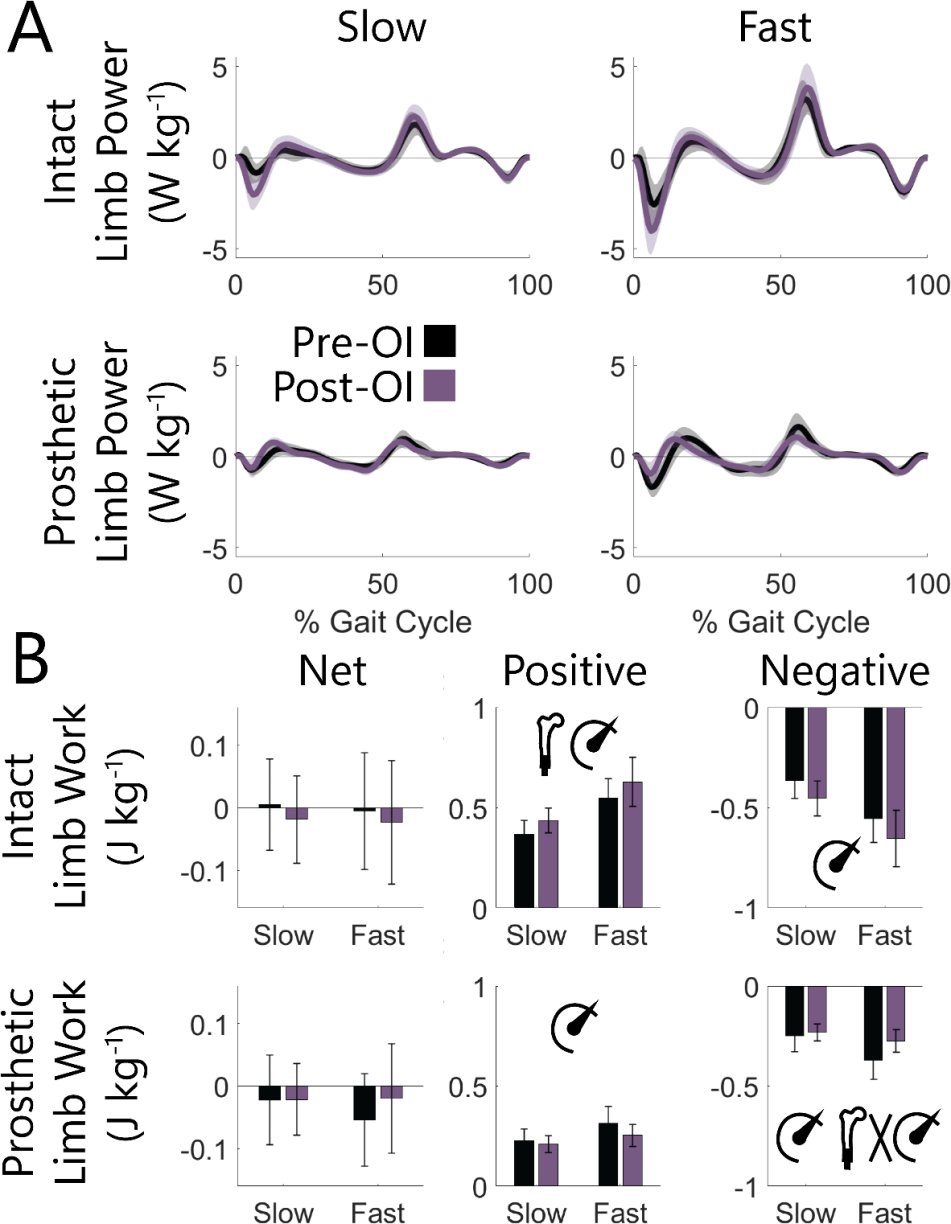
(**A**) Across-participant ensemble averaged limb powers and (**B**) mean limb work values across slow (0.6–1.1 m s^− 1^) and fast (1.1–1.6 m s^− 1^) walking speeds for socket and OI interfaces. Shaded regions and error bars represent ± 1 s.d. Values were normalized to each participant’s body mass. Significant effects (*P* < 0.050) of interface, speed, and interface-speed interaction were determined using two-way repeated measures ANOVAs and are graphically represented as an osseointegrated femur, speedometer, and their interaction. Statistical results of the effect of limb within each interface and pairwise comparisons are reported in text.

While limb powers exhibited larger peaks on the intact vs. prosthetic limbs for both interfaces, particularly at fast speeds (Fig. 2A), there was no difference in net limb work between limbs (i.e., prosthetic vs. intact; *P* > 0.496) or a limb-speed interaction (*P* > 0.479) for both interfaces (Fig. 2B). Similarly, comparison of net limb work between interfaces within each limb showed no effect of interface (*P* > 0.456), speed (*P* > 0.197), or their interaction (*P* > 0.087). Positive and negative work components generally supported the observation of larger peak powers on the intact limb and smaller peaks on the prosthetic limb with an OI vs. socket interface, particularly at fast speeds (Fig. 2A). On the intact limb, there was 0.07 J kg^− 1^ more positive (*P* = 0.045), but not significantly more negative (*P* = 0.108) limb work with an OI vs. socket interface. On the prosthetic limb, there was an interface-speed interaction (*P* = 0.021); there was 0.10 J kg^− 1^ less negative limb work (*P* = 0.039) during fast walking speeds with an OI vs. socket interface. A similar trend toward less prosthetic positive limb work during fast walking speeds with an OI vs. socket interface did not reach significance (interface-speed interaction *P* = 0.052). Fast vs. slow walking speeds were associated with larger positive and negative work values on both limbs (*P* < 0.001).

Socket compression during load acceptance resulted in a burst of negative power (Fig. 4A), which corresponded to 0.14 J kg^− 1^ of negative work at slow speeds and 0.17 J kg^− 1^ at fast speeds (*P* < 0.001, Fig. 4B). This negative work was not returned over the remainder of stride as similar quantities of positive work (slow: 0.07 J kg^− 1^, fast: 0.10 J kg^− 1^, speed effect: *P* = 0.005).

**Fig. 3:**
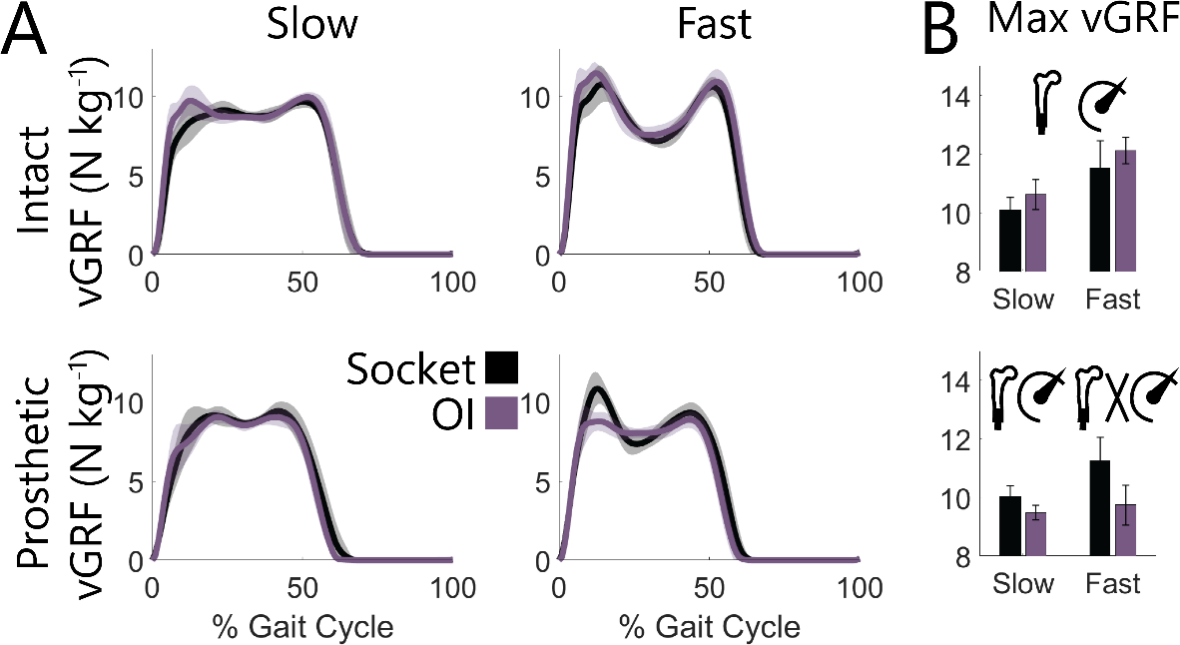
Across-participant (**A**) ensemble averaged vertical ground reaction forces (vGRFs) and (**B**) maximum vGRFs across slow (0.6–1.1 m s^− 1^) and fast (1.1–1.6 m s^− 1^) walking speeds for socket and OI interfaces. Shaded regions and error bars represent ± 1 s.d. Values were normalized to each participant’s body mass. Significant effects (*P* < 0.050) of interface, speed, and interface-speed interaction were determined using two-way repeated measures ANOVAs and are graphically represented as an osseointegrated femur, speedometer, and their interaction. Statistical results of pairwise comparisons are reported in text.

On the intact limb, maximum vertical ground reaction forces (vGRFs; Fig. 3A) were 0.55 N kg^− 1^ larger with an OI vs. socket interface (*P* = 0.010; Fig. 3B). On the prosthetic limb, maximum vGRFs were 1.03 N kg^− 1^ smaller with an OI vs. socket interface (*P* = 0.005) – a difference which was exacerbated at fast speeds (slow: 0.55 N kg^− 1^ less, *P* = 0.012; fast: 1.50 N kg^− 1^ less, *P* = 0.006). Fast speeds were associated with larger maximum vGRFs than slow speeds on both limbs (*P* < 0.005). Anteroposterior GRFs (Fig. S1) demonstrated that on the intact limb early stance posterior GRFs were larger with an OI vs. socket interface across speeds (*P* = 0.045) while late stance posterior GRFs were not affected by interface (*P* = 0.578, interface-speed interaction: *P* = 0.399). On the prosthetic limb, posterior GRFs demonstrated an interface-speed interaction (*P* = 0.016), where during fast walking posterior GRFs tended to be 0.37 N kg^− 1^ smaller with an OI vs. socket interface (*P* = 0.066). Anterior GRFs were 0.18 N kg^− 1^ larger on the prosthetic limb with an OI vs. socket interface across speeds (*P* = 0.006). Fast vs. slow speed increased both posterior and anterior GRFs for both limbs (*P* < 0.001).

### Osseointegration does not change positive prosthetic limb hip work but does result in less negative hip work, particularly at faster walking speeds

**Fig. 4:**
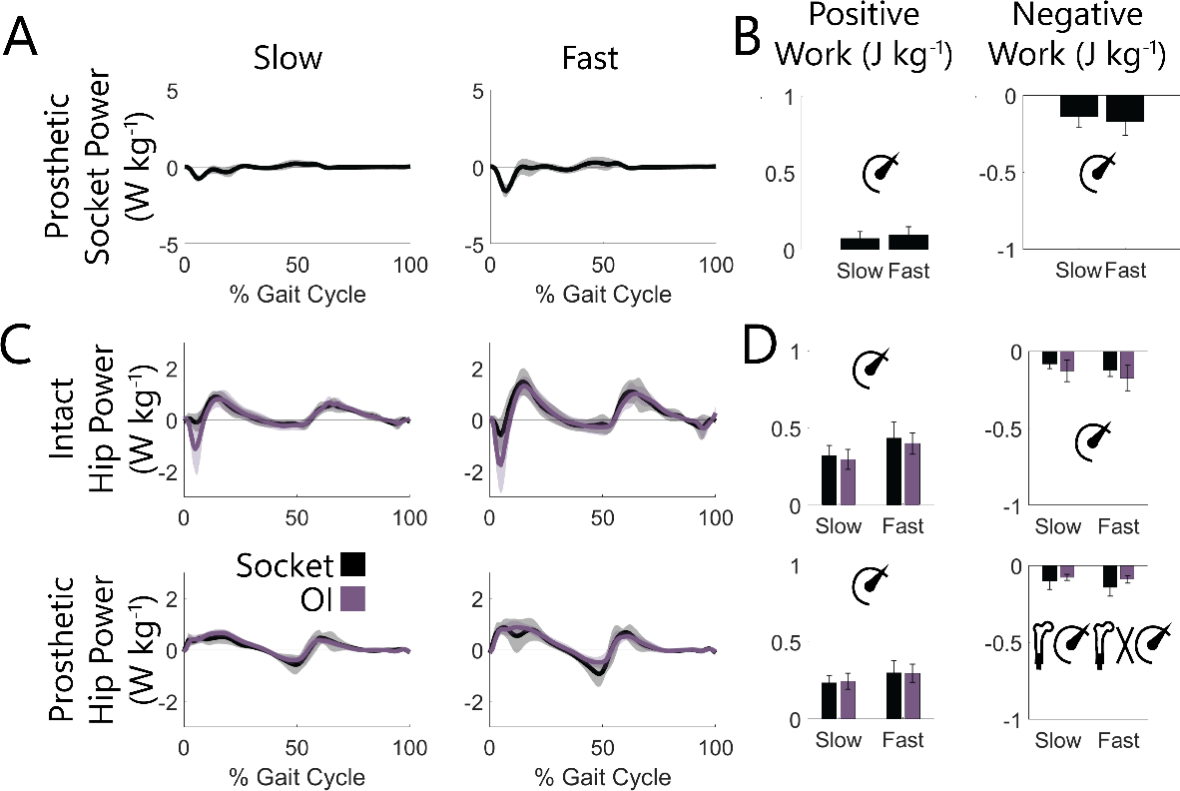
Across-participant ensemble averaged (**A**) socket and (**C**) hip powers with (**B**, **D**) associated positive and negative mean work values across slow (0.6–1.1 m s^− 1^) and fast (1.1–1.6 m s^− 1^) walking speeds for socket and OI interfaces, where applicable. Hip power was summed across all 3 degrees of freedom of the hip joint. Shaded regions and error bars represent ± 1 s.d. Values were normalized to each participant’s body mass. Significant effects (*P* < 0.050) of interface, speed, and interface-speed interaction were determined using two-way repeated measures ANOVAs and are graphically represented as an osseointegrated femur, speedometer, and their interaction. Statistical results of pairwise comparisons are reported in text. Socket work values were compared between speeds using pairwise t-tests.

**Fig. 5:**
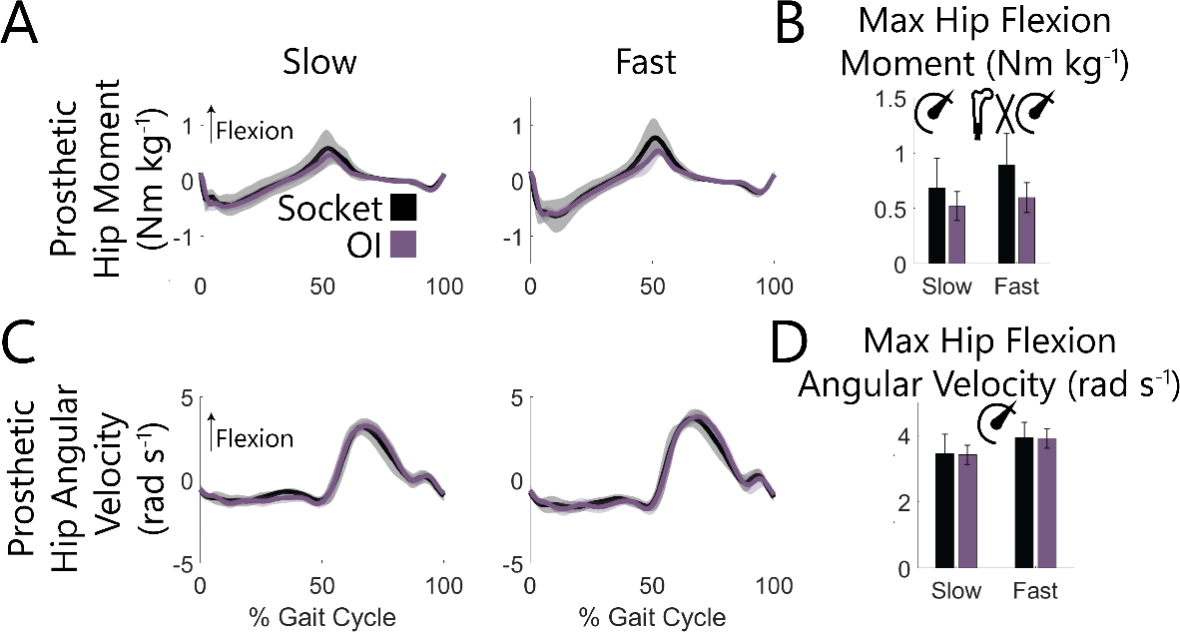
Across-participant prosthetic side ensemble averaged (**A**) sagittal hip moments and (**C**) sagittal hip angular velocities with (**B**, **D**) associated maxima at slow (0.6–1.1 m s^− 1^) and fast (1.1–1.6 m s^− 1^) walking speeds for socket and OI interfaces. Shaded regions and error bars represent ± 1 s.d. Moments were normalized to each participant’s body mass. Significant effects (*P* < 0.050) of interface, speed, and interface-speed interaction were determined using two-way repeated measures ANOVAs and are graphically represented as an osseointegrated femur, speedometer, and their interaction. Statistical results of pairwise comparisons are reported in text.

At the hip of both the prosthetic and intact limbs, periods of positive power were similar with an OI vs. socket interface (Fig. 4C). Consistent with that observation, speed (*P* < 0.001), but not interface (*P* > 0.443), was associated with more positive hip work across both limbs (Fig. 4D). For the intact limb, negative hip work was also only affected by speed (*P* < 0.001), and not interface (*P* = 0.197), while on the prosthetic limb there was 0.04 J kg^− 1^ less negative hip work with an OI vs. socket interface (*P* = 0.044) – a difference which was exacerbated at fast speeds (interface-speed interaction *P* < 0.001; slow: 0.03 J kg^− 1^ less *P* = 0.162, fast: 0.05 J kg^− 1^ less *P* = 0.012). This decrease in negative hip work resulted from a smaller peak hip flexion moment in late stance with an OI vs. socket interface (*P* = 0.063), particularly during fast speeds (interface-speed interaction *P* = 0.004, slow speed OI vs. socket *P* = 0.153, fast speed OI vs. socket *P* = 0.029), as opposed to a difference in angular velocity (interface *P* = 0.879, interface-speed interaction *P* = 0.950; Fig. 5).

There were no significant effects of interface or interface-speed interactions for both intact and prosthetic knee positive and negative work (*P* > 0.072; Supplementary Fig. S2). At the ankle-foot complexes (Supplementary Fig. S3), on the intact limb there was 0.05 J kg^− 1^ more positive ankle-foot work (*P* = 0.026), while on the prosthetic limb there was 0.06 J kg^− 1^ more negative work with an OI vs. socket interface (*P* = 0.047). Sagittal hip, knee, and ankle kinematics are included as Supplementary Fig. S4, S5, and S6, respectively.

## Discussion

The overall goal of this study was to investigate the effects of prosthesis interface (OI vs. socket) on limb and joint level mechanical energetics during walking in persons with unilateral transfemoral limb loss. We used custom musculoskeletal models of unilateral transfemoral limb loss with socket-based suspension and OI, together with full-body biomechanical data collected longitudinally before (using socket-based prostheses) and 24-months after OI, to test three hypotheses: H1) the net work of the prosthetic and intact limbs over a stride would be zero with both interfaces, H2) a shift in load distribution from the prosthetic toward the intact limb with an OI interface would lead to larger mechanical work demands on the intact limb and smaller demands on the prosthetic limb, and H3) there would be a decrease in positive work demand on the hip of the prosthetic limb with an OI interface.

H1 was supported by minimal net work being performed on the whole-body COM by the prosthetic and intact limbs with both interfaces. Here, mean work magnitudes across speeds, interfaces, and limbs ranged between 0.01 and 0.05 J kg^− 1^, which are similar to the mean net limb work over a stride of 0.01 J kg^− 1^ for uninjured individuals^16^. Methodologically, this alignment suggests that our kinematics-based estimates of COM velocity do not exhibit biased phase shifts relative to GRF that deviate from GRF-based COM velocity estimates^26^. A lack of appreciable net positive or negative work performed by the prosthetic or intact limbs is similar to what has been observed during walking among individuals with unilateral transtibial amputation^21^, and indicates there is no across-participant strategy of using one limb as a net energy “source” and the other as a “sink” on a whole-stride basis. Maintaining zero net work by each limb demonstrates individuals with limb loss maintain GRF and COM velocity trajectories with a relative phase that results in pendular mechanics of each limb, despite asymmetries in force production between limbs. This contrasts with strategies used by stroke survivors, where the paretic and non-paretic limbs produce net positive and net negative work, respectively^22,23^. This may suggest that individuals with lower limb amputation execute walking with pendular limb mechanics to a greater extent than other populations with lateralized impairments, which also remains the case 2 years after OI.

H2 was also supported by a 9.7% decrease in peak vGRF on the prosthetic limb and a 5.1% increase for the intact limb observed with an OI vs. socket interface. This prosthetic limb offloading strategy is consistent with most, but not all^24^, prior longitudinal and cross sectional studies, which have demonstrated peak forces of ~ 80% of bodyweight post-OI relative to peak forces of ~ 100% bodyweight using socket suspension, with this effect also being independent of differences in self-selected walking speed^11,27–31^. This offloading of the osseointegrated limb seemingly contrasts with increases in mobility and prosthesis embodiment reported by individuals with an OI interface, which should occur with increased trust in the prosthesis^7–10^. One mechanism that could explain this decreased peak loading is the sensitivity of osseointegrated bone to external loads and vibrations^12,32,33^. Given that increased intact limb loading is already a concern for the long-term joint health of individuals with unilateral lower limb loss^34–36^, future studies should consider investigating (1) whether increased rates of joint degeneration are present after OI, and (2) whether attenuation of vibrations on the osseointegrated limb normalizes loads between limbs.

Consistent with H2, the mechanical energetic effects of a shift in loading toward the intact limb were increases in intact limb collision and push-off work, in addition to decreases in prosthetic limb collision and push-off. In able-bodied individuals, inducing similar effects by enforcing step time asymmetries results in an increased metabolic cost of walking^37^. However, comparison of the metabolic cost of walking at self-selected walking speeds post-OI, where self-selected speeds were not different with an OI vs. socket interface, has shown reductions in oxygen consumption and physiological cost index as soon as 1-year post-OI^3,6,14^. This discrepancy highlights the limited ability of overall limb mechanics to capture differences in muscle dynamics that directly drive metabolic cost differences, particularly in populations that contend with increased co-contraction^38–41^, and hence the necessity of direct muscle imaging and electromyography driven simulations.

Our hypothesis (H3) that positive work demands on the hip of the prosthetic limb would decrease with removal of the socket interface was not supported. While net socket losses were appreciable (~ 6 J estimated per stride across both speeds), the elimination of those losses with an OI interface did not produce a marked difference in positive prosthetic limb hip power coinciding with hip extension in early prosthetic stance, and there were no differences in positive hip work on the prosthetic side. Taking these results together with the magnitudes of decreased negative work at the overall prosthetic limb with an OI interface (~ 2 J and ~ 8 J during slow and fast walking, respectively), and the relative invariance in powers of prosthetic joints with OI vs. socket interfaces, indicates that removed socket losses were reflected at the COM level, as opposed to leading to a redistribution of power within the prosthetic limb. Discrepancies in accounting for differences between summed prosthetic joint and overall prosthetic limb work may also point to potential alterations in trunk and soft-tissue mechanics post-OI^42–44^. On the prosthetic limb, the most notable change in joint mechanical energetics with an OI interface was a reduction in negative hip work resulting from a decreased late stance hip flexion moment, which is consistent with previous findings^24^. We found that this decreased moment was due to a combined decrease in prosthetic limb vertical GRF and an increased anterior GRF in late stance with an OI interface. A more anteriorly directed GRF can be explained by increased hip extension increasing trailing limb angle, which may have been facilitated by the increased hip range of motion made possible without socket suspension^2,45^.

This study had several important limitations. Our relatively small set of participants consisted of military service members and were thus predominantly younger (mean age: 40 years) males (7/8) with traumatic amputations. This contrasts with the general population of individuals with lower limb loss, which is older, more equally distributed by sex, and more prone to dysvascular amputation etiologies^46,47^. Additionally, although the within-subject, longitudinal design is beneficial for isolating sources of variability, the primary inclusion criterion of a poor fitting socket may bias our findings toward larger relative socket motion. Thus, our socket interface data may not be representative of the larger unilateral transfemoral population. Another limitation that may confound a direct link between an increased limb offloading strategy and OI is that the same implant system (OPRA) and rehabilitation protocol template were used for all participants. All participants received rehabilitation at the same hospital using a similar protocol which progressively increased loading of the abutment. Phase 1 of rehabilitation (> 4 weeks after the second stage operation) involved loading the abutment to 20 kg of bodyweight and increasing this load by no more than 10 kg every week until 40 kg was reached. Phases 2 (> 7 weeks post-op), 3, and 4 (> 11 weeks post-op) involved reaching abutment loads of 50, 80, and 100% of bodyweight, after which milestones transitioned to ambulating with crutches (> 14 weeks post-op) and finally independent ambulation (> 16 weeks post-op). However, we contend the specifics of this protocol are a minor limitation since prosthetic limb offloading post-OI is consistent with literature across different implant systems and rehabilitation protocols. An important limitation of our modeling approach was that socket pistoning limited the motion of the socket to the long axis of the femur. We selected this degree of freedom to limit the uncertainty introduced by using a global inverse kinematics-based estimate of relative socket-femur motion and because the axial direction is the degree of freedom under the largest load. We have confidence in our estimates of relative motion since our estimates of the range of axial socket-femur displacement femur at both slow (3.1 cm) and fast (3.4 cm) speeds were of a similar magnitude as literature values (~ 1.6 cm)^13,14^, and participants who are eligible for OI may be expected to have larger socket-femur motion compared to the general population of individuals with transfemoral amputation. Additionally, the prosthetic ankle-foot joint was limited to sagittal angular motion and vertical displacement about an approximated prosthetic joint center of one prosthetic foot type, which may have limited the ability of the model to fully capture different prosthetic ankle-foot dynamics. However, this limitation was also partially addressed by using six degree of freedom estimates of ankle-foot power, and individual limb power estimates being agnostic to the mechanics of individual joints.

In summary, this is the first study to investigate the mechanical energetic implications of transitioning from a socket-suspended to osseointegrated prosthesis among individuals with unilateral transfemoral limb loss. We identified that with both socket and OI interfaces the net mechanical energy demands on the prosthetic and intact limbs are similar, while a strategy of shifting weight toward the intact limb with an OI interface leads to larger positive and negative intact limb work in addition to smaller positive and negative prosthetic limb work. We also found that removal of a socket interface is associated with less negative prosthetic limb work overall, as opposed to reducing demands on the hip joint of the prosthetic limb. Our findings highlight the need to investigate the mechanisms of an intact limb overuse strategy, in addition to the differences in muscle mechanics post-OI that may lead to more efficient gait despite such increased asymmetries. Optimizing OI rehabilitation to ensure patient comfort with large abutment loads while also building trust in their osseointegrated prosthesis may be critical for minimizing the risk of long-term joint degeneration in this population.

## Materials and methods

### Participants

This analysis was performed on biomechanical data being collected as part of a clinical trial longitudinally evaluating individuals with transfemoral limb loss who underwent a femoral osseointegration procedure using the two-stage OPRA system (Integrum, Mölndal, Sweden)^48^. For the larger study, participants were included if they were military beneficiaries between 22 and 65 years of age with transfemoral limb loss and had difficulties wearing a traditional socket-suspended prosthesis. For this analysis, a subset of eight participants with unilatateral transfemoral limb loss were selected based on biomechanical gait evaluations completed (without use of assistive devices) using a socket-suspended prosthesis (suction), and ~ 24 months post-OI, using a prosthesis affixed to the femoral abutment (Table 1). This sample size was supported based on a comparison of prosthetic limb loading during walking before and after transfemoral OI^11^, which indicated a sample of seven participants would be capable of detecting a significant (*p* < 0.05) effect of OI on prosthetic limb vertical GRF with 95% power. For the biomechanical gait evaluation, participants walked overground on a 15-meter walkway at each of three targeted speeds: 0.7 m s^− 1^, 1.0 m s^− 1^, and 1.4 m s^− 1^, when possible. Walking speeds were enforced using auditory feedback of the velocity of a marker placed on the participant’s jugular notch. All participants provided written informed consent as approved by the Walter Reed National Military Medical Center Institutional Review Board (Protocol WRNMMC-2017-0091). All methods were carried out in accordance with relevant guidelines and regulations.

**Table 1:** Participant demographics. All participants used an ischial containment socket prior to osseointegration. Residual femur lengths were calculated as the linear distance between the femoral head and the most distal cortical bone of the residual femur from DEXA images.

					Socket Interface				OI Interface			
Participant	Sex	Stature (cm)	Age at Socket Interface Session (yrs)	Time Since Amputation (mo)	Residual Femur Length (cm)	Body Mass with Prosthesis (kg)	Prosthetic Foot	Prosthetic Knee	Residual Femur Length (cm)	Body Mass with Prosthesis (kg)	Prosthetic Foot	Prosthetic Knee
1	M	170.2	47	86	23	83.1	Freedom Hydraulic	Ottobock X3	21	85.4	Hydraulic Kinterra	Ottobock X3
2	M	150.5	29	34	24	53.9	Ottobock C-Walk	Ottobock X3	24	52.4	Blatchford Echelon	Ottobock X3
3	M	176.5	27	79	33	82.0	Fillauer Wave Allsport	Ottobock X3	28	82.6	Fillauer Allpro	Ottobock X3
4	M	179.5	51	56	32	88.7	Ossur Proflex	Ottobock X3	28	89.1	Fillauer Allpro	Ottobock X3
5	M	193.0	28	102	27	105.3	Ossur Variflex	Ossur Rheo	25	106.6	Ossur Proflex	Ottobock X3
6	M	179.0	33	117	27	73.0	College Park Soleus Tactical	Ottobock X3	26	71.4	Ossur Proflex Pivot	Ottobock X3
7	F	165.5	61	49	30	75.0	Not Reported	Ossur Rheo	24	81.1	Ossur Proflex Pivot	Ottobock X3
8	M	178.5	45	157	30	115.1	Ossur Variflex	Ottobock X3	28	96.1	Not Reported	Ottobock X3

### Musculoskeletal modeling

Generic models representing unilateral transfemoral limb loss with a socket-based prosthesis (Fig. 1A) and an osseointegrated prosthesis (Fig. 1B) were developed in OpenSim 4.4^25^. Both models were modified versions of a full-body musculoskeletal model of an uninjured individual^49^, with several modifications: (1) the residual femur was cut to 50% of the intact femur length, (2) a prosthetic knee was included, representing the Ottobock X3 (Duderstadt, Germany), with proximal and distal segments connected at a rotational degree of freedom, (3) a representative energy storing and returning prosthetic foot was included (College Park Soleus Tactical; Warren, MI, USA), with proximal and distal segments connected about a rotational degree of freedom and a proximal-distal translational degree of freedom, (4) a pylon segment was included between the prosthetic knee and foot, and (5) a pylon segment was included connecting the prosthetic knee to proximal structures. For the socket-based prosthesis, an additional component of a generic transfemoral socket was developed in SolidWorks 2023 (Dassault Systèmes, Vélizy-Villacoublay, France) based on measurements and geometry of an ischial containment socket. One translational degree of freedom was included to represent “pistoning” motion of the socket relative to the residual femur. For the osseointegrated prosthesis, additional modeled components included the titanium abutment within the femur (Integrum, Mölndal, Sweden) and a failsafe device (OPRA Axor II, Integrum, Mölndal, Sweden) between the abutment and the pylon proximal to the prosthetic knee, both developed in SolidWorks 2023. Mass properties of the residual femur were approximated based on literature^50^, and properties of componentry were estimated using SolidWorks 2023. Although muscle-tendon level estimates were not used in this work, modeled muscle-tendon paths were edited such that the distal attachment points were 1 cm proximal to the distal end of the residual femur^51^.

### Kinematics and kinetics

For each participant, all model segment geometries and masses were scaled using within-session static standing trials, full-body X-ray images (Discovery A; Hologic, Marlborough, MA, USA), and body mass measurements. Note that through this scaling process, the residual femur length (i.e., 50% of intact femur length) in the generic model was scaled to each participant’s residual femur length based on X-ray images. During walking, GRFs were collected at 1200 Hz from six in-ground force platforms (AMTI, Watertown, MA, USA) and trajectories of 51 reflective markers (modified Cleveland Clinic marker set) were tracked at 120 Hz using an 18-camera motion capture system (Qualysis, Gotenburg, Sweden). All strides where forces acting on a limb were fully attributable to a single force platform were manually identified and segmented using a 40 N threshold in vertical GRF (268 and 282 total strides for the intact and prosthetic limbs, respectively, across all participants). Joint angles were calculated in OpenSim, using a global least-squares fit between model-fixed markers and experimental marker trajectories. This global least-squares approach allows for estimation of residual femur kinematics among individuals using socket interfaces by incorporating both socket-fixed marker trajectories in addition to assuming limited model degrees of freedom (i.e., only socket pistoning at the residual femur and socket interface;^52^). Kinematics and GRFs were low-pass filtered using 4th order zero-phase Butterworth filters with cutoff frequencies of 6 and 15 Hz, respectively, prior to subsequent analyses. Joint moments were calculated using the OpenSim Inverse Dynamics tool using both inverse kinematics solutions and GRFs applied to the calcanei or the distal segment of the prosthetic foot for intact/prosthetic strides, respectively. Whole-body COM and segmental kinematics were calculated using inverse kinematics solutions and the OpenSim Body Kinematics tool.

### Limb and joint mechanical energetics

Rotational joint mechanical powers were calculated as the products of joint velocities and joint moments. Translational joint mechanical powers were calculated as the products of joint forces and linear velocities. Both prosthetic and intact ankle-foot powers were calculated using a unified deformable segment approach lumping all segments distal to the shank^53^. Limb mechanical powers were calculated as the dot products of GRFs and COM velocity summed with the peripheral powers of each limb segment moving relative to the whole-body COM^16,26,54^. Peripheral powers were calculated by summing the time derivatives of the rotational and translational components of kinetic energy of the limb segments^55,56^. Mechanical work was calculated by integrating mechanical power with respect to time, with positive or negative work components being calculated by integrating exclusively positive or negative powers, respectively.

### Statistical analyses

Statistical analyses were performed in Matlab R2022a (Mathworks, Natick, MA, USA). Walking speed was calculated post-hoc for each stride as the mean velocity of the whole-body COM in the anteroposterior direction. Stride speeds were binned into slow (0.6–1.1 m s^− 1^, mean ± standard deviation: 0.94 ± 0.12 m s^− 1^) and fast (1.1–1.6 m s^− 1^, 1.32 ± 0.12 m s^− 1^) categories. For comparisons between prosthetic and intact limbs, two-way repeated measures ANOVAs were run with main effects of limb, speed bin, and the interaction effect of limb and speed bin. Similarly, for comparisons between socket and OI interfaces, repeated measures ANOVAs were run with main effects of interface, speed bin, and the interaction effect of interface and speed bin. Normality was confirmed for all results (absolute skewness < 1.7, absolute kurtosis < 3.6),^57^ aside from intact limb negative hip work (kurtosis = 6.29) and positive socket work (kurtosis = 4.78), for which there were interpreted significant effects of interface. Two-tailed post-hoc paired t-tests were performed with Bonferroni correction. A paired t-test was also used to compare socket work between speeds. Statistical significance was concluded for *P* < 0.050.

## Electronic supplementary material

Below is the link to the electronic supplementary material.

Supplementary Material 1

## Data Availability

The datasets generated and/or analyzed for the current study are available at: https://zenodo.org/doi/10.5281/zenodo.13646210.

## References

[1] Zaid, M. B., O’Donnell, R. J., Potter, B. K. & Forsberg, J. A. Orthopaedic osseointegration: state of the art. *J. Am. Acad. Orthop. Surg.* **27**, E977. 31181031 10.5435/JAAOS-D-19-00016

[2] Hagberg, K., Häggström, E., Uden, M. & Brånemark, R. Socket versus bone-anchored trans-femoral prostheses: hip range of motion and sitting comfort. *Prosthet. Orthot. Int.* **29**, 153–163 (2005). 16281724 10.1080/03093640500238014

[3] Van De Meent, H., Hopman, M. T. & Frölke, J. P. Walking ability and quality of life in subjects with transfemoral amputation: A comparison of osseointegration with socket prostheses. *Arch. Phys. Med. Rehabil*. **94**, 2174–2178 (2013). 23774380 10.1016/j.apmr.2013.05.020

[4] Leijendekkers, R. A. et al. Functional performance and safety of bone-anchored prostheses in persons with a transfemoral or transtibial amputation: a prospective one-year follow-up cohort study. *Clin. Rehabil*. **33**, 450–464 (2019). 30537856 10.1177/0269215518815215 PMC6416705

[5] Hagberg, K. et al. A 15-year follow-up of transfemoral amputees with bone-anchored transcutaneous prostheses. *Bone Joint J.* **102-B**, 55–63 (2020). 10.1302/0301-620X.102B1.BJJ-2019-0611.R1 31888375

[6] Hagberg, K., Hansson, E. & Brånemark, R. Outcome of percutaneous osseointegrated prostheses for patients with unilateral transfemoral amputation at two-year follow-up. *Arch. Phys. Med. Rehabil*. **95**, 2120–2127 (2014). 25064778 10.1016/j.apmr.2014.07.009

[7] Davis-Wilson, H. C. et al. Improvements in disability and function in people with lower-limb amputation one year after prosthesis osseointegration. *Prosthet. Orthot. Int.* **47**, 343–349 (2023). 36701203 10.1097/PXR.0000000000000200

[8] Al Muderis, M., Lu, W. & Li, J. J. Das osseointegrated prosthetic limb Zur behandlung von amputationen der unteren extremitäten: erfahrungen und ergebnisse. *Unfallchirurg* **120**, 306–311 (2017). 28070628 10.1007/s00113-016-0296-8

[9] Gaffney, B. M. et al. Daily steps and stepping cadence increase one-year following prosthesis osseointegration in people with lower-limb amputation. *Disabil. Rehabil*. **46**, 1432–1437 (2024). 37073780 10.1080/09638288.2023.2200036 PMC10584988

[10] Lundberg, M., Hagberg, K. & Bullington, J. My prosthesis as a part of me: A qualitative analysis of living with an osseointegrated prosthetic limb. *Prosthet. Orthot. Int.* **35**, 207–214 (2011). 21697203 10.1177/0309364611409795

[11] Blumentritt, S., Schmalz, T., Layher, F., Timmermann, A. & Aschoff, H. H. Force transmission capacity of the lower limb during walking of amputees with bone-anchored prostheses compared with socket prostheses and persons with hip replacements. *Clin. Biomech.* **110** (2023). 10.1016/j.clinbiomech.2023.106099 37832468

[12] Häggström, E., Hagberg, K., Rydevik, B. & Brånemark, R. Vibrotactile evaluation: osseointegrated versus socket-suspended transfemoral prostheses. *J. Rehabil Res. Dev.* **50**, 1423–1434 (2013). 24699977 10.1682/JRRD.2012.08.0135

[13] Maikos, J. T., Chomack, J. M., Loan, J. P., Bradley, K. M. & D’Andrea, S. E. Effects of prosthetic socket design on residual femur motion using dynamic stereo X-Ray - A preliminary analysis. *Front. Bioeng. Biotechnol.* **9**, (2021). 10.3389/fbioe.2021.697651 PMC8383143 34447740

[14] Kooiman, V., Haket, L., Verdonschot, N., Leijendekkers, R. & Weerdesteyn, V. Oxygen consumption and gait dynamics in transfemoral bone-anchored prosthesis users compared to socket-prosthesis users: A cross-sectional study. *Gait Posture*. **103**, 12–18 (2023). 37075553 10.1016/j.gaitpost.2023.04.008

[15] Jaegers, S. M., Arendzen, J. H. & De Jongh, H. J. Prosthetic Gait of Unilateral Transfemoral Amputees: A Kinematic Study*. Arch. Phys. Med. Rehabil. ***76**, 736–743 (1995). 10.1016/s0003-9993(95)80528-1 7632129

[16] Zelik, K. E., Takahashi, K. Z. & Sawicki, G. S. Six degree-of-freedom analysis of hip, knee, ankle and foot provides updated Understanding of Biomechanical work during human walking. *J. Exp. Biol.* **218**, 876–886 (2015). 25788726 10.1242/jeb.115451

[17] Donelan, J. M., Kram, R. & Kuo, A. D. Mechanical work for step-to-step transitions is a major determinant of the metabolic cost of human walking. *J. Exp. Biol.* **205**, 3717–3727 (2002). 12409498 10.1242/jeb.205.23.3717

[18] Russell Esposito, E., Rábago, C. A. & Wilken, J. The influence of traumatic transfemoral amputation on metabolic cost across walking speeds. *Prosthet. Orthot. Int.* **42**, 214–222 (2018). 28655287 10.1177/0309364617708649

[19] Adamczyk, P. G. & Kuo, A. D. Mechanisms of gait asymmetry due to Push-Off deficiency in unilateral amputees. *IEEE Trans. Neural Syst. Rehabil. Eng.* **23**, 776–785 (2015). 25222950 10.1109/TNSRE.2014.2356722 PMC4483155

[20] Kuo, A.D., Donelan, J.M., & Ruina, A. Energetic Consequences of Walking Like an Inverted Pendulum: Step-to-Step Transitions*.**Exerc. Sport Sci. Rev.* **33**, 88–97 (2005). 10.1097/00003677-200504000-00006 15821430

[21] Houdijk, H., Pollmann, E., Groenewold, M., Wiggerts, H. & Polomski, W. The energy cost for the step-to-step transition in amputee walking. *Gait Posture*. **30**, 35–40 (2009). 19321343 10.1016/j.gaitpost.2009.02.009

[22] Mahon, C. E., Farris, D. J., Sawicki, G. S. & Lewek, M. D. Individual limb mechanical analysis of gait following stroke. *J. Biomech.* **48**, 984–989 (2015). 25698237 10.1016/j.jbiomech.2015.02.006

[23] McCain, E. M. et al. Mechanics and energetics of post-stroke walking aided by a powered ankle exoskeleton with speed-adaptive myoelectric control. *J. Neuroeng. Rehabil* **16**, (2019). 10.1186/s12984-019-0523-y PMC6521500 31092269

[24] Vandenberg, N. W. et al. Unilateral transfemoral osseointegrated prostheses improve joint loading during walking. *J. Biomech.* **155**, (2023). 10.1016/j.jbiomech.2023.111658 PMC10330663 37276681

[25] Delp, S. L. et al. OpenSim: Open-Source software to create and analyze dynamic simulations of movement. *IEEE Trans. Biomed. Eng.* **54**, 1940–1950 (2007). 18018689 10.1109/TBME.2007.901024

[26] Donelan, J. M., Kram, R. & Kuo, A. D. Simultaneous positive and negative external mechanical work in human walking. *J. Biomech.* **35**, 117–124 (2002). 11747890 10.1016/s0021-9290(01)00169-5

[27] Stenlund, P. et al. Effect of load on the bone around bone-anchored amputation prostheses. *J. Orthop. Res.* **35**, 1113–1122 (2017). 27341064 10.1002/jor.23352

[28] Lee, W. C. C. et al. Magnitude and variability of loading on the osseointegrated implant of transfemoral amputees during walking. *Med. Eng. Phys.* **30**, 825–833 (2008). 17977050 10.1016/j.medengphy.2007.09.003

[29] Thesleff, A. et al. Loads at the Implant-Prosthesis interface during free and aided ambulation in osseointegrated transfemoral prostheses. *IEEE Trans. Med. Robot Bionics*. **2**, 497–505 (2020).

[30] Frossard, L., Häggström, E., Hagberg, K. & Brånemark, R. Load applied on bone-anchored transfemoral prosthesis: characterization of a prosthesis-A pilot study. *J. Rehabil Res. Dev.* **50**, 619–634 (2013). 24013910 10.1682/jrrd.2012.04.0062

[31] Morgenroth, D. C., Roland, M., Pruziner, A. L. & Czerniecki, J. M. Transfemoral amputee intact limb loading and compensatory gait mechanics during down slope ambulation and the effect of prosthetic knee mechanisms. *Clin. Biomech.* **55**, 65–72 (2018). 10.1016/j.clinbiomech.2018.04.007 29698851

[32] Habre-Hallage, P. et al. Brain plasticity and cortical correlates of osseoperception revealed by punctate mechanical stimulation of osseointegrated oral implants during FMRI. *Eur. J. Oral Implantol.* **5**, 175–190 (2012). 22866293

[33] Lundborg, G., Waites, A., Björkman, A., Rosén, B. & Larsson, E. M. Functional magnetic resonance imaging shows cortical activation on sensory stimulation of an osseointegrated prosthetic thumb. *Scand. J. Plast. Reconstr. Surg. Hand Surg.* **40**, 234–239 (2006). 16911998 10.1080/02844310600787005

[34] Norvell, D. C. et al. The prevalence of knee pain and symptomatic knee osteoarthritis among veteran traumatic amputees and nonamputees. *Arch. Phys. Med. Rehabil*. **86**, 487–493 (2005). 15759233 10.1016/j.apmr.2004.04.034 PMC11803826

[35] Struyf, P. A., van Heugten, C. M., Hitters, M. W. & Smeets, R. J. The prevalence of osteoarthritis of the intact hip and knee among traumatic leg amputees. *Arch. Phys. Med. Rehabil*. **90**, 440–446 (2009). 19254609 10.1016/j.apmr.2008.08.220

[36] Morgenroth, D. C., Gellhorn, A. C. & Suri, P. Osteoarthritis in the Disabled Population: A Mechanical Perspective. *PM&R ***4**, S20–S27 (2012). 10.1016/j.pmrj.2012.01.003 22632698

[37] Ellis, R. G., Howard, K. C. & Kram, R. The metabolic and mechanical costs of step time asymmetry in walking. *Proceedings of the Royal Society B: Biological Sciences* **280** (2013). 10.1098/rspb.2012.2784 PMC3574372 23407831

[38] Quesada, R. E., Caputo, J. M. & Collins, S. H. Increasing ankle push-off work with a powered prosthesis does not necessarily reduce metabolic rate for transtibial amputees. *J. Biomech.* **49**, 3452–3459 (2016). 27702444 10.1016/j.jbiomech.2016.09.015

[39] Caputo, J. M. & Collins, S. H. Prosthetic ankle push-off work reduces metabolic rate but not collision work in non-amputee walking. *Sci. Rep.* **4**, (2014). 10.1038/srep07213 PMC4252906 25467389

[40] Piche, E. et al. Metabolic cost and co-contraction during walking at different speeds in young and old adults. *Gait Posture*. **91**, 111–116 (2022). 34673446 10.1016/j.gaitpost.2021.10.014

[41] Ortega, J. D. & Farley, C. T. Individual limb work does not explain the greater metabolic cost of walking in elderly adults. *J. Appl. Physiol.* **102**, 2266–2273 (2007). 17363623 10.1152/japplphysiol.00583.2006

[42] Gaffney, B. M., Thomsen, P. B., Leijendekkers, R. A., Christiansen, C. L. & Stoneback, J. W. Lumbopelvic movement coordination during walking improves with transfemoral bone anchored limbs: implications for low back pain. *Gait Posture*. **109**, 318–326 (2024). 38432038 10.1016/j.gaitpost.2024.02.015 PMC11015906

[43] Zelik, K. E. & Kuo, A. D. Human walking isn’t all hard work: evidence of soft tissue contributions to energy dissipation and return. *J. Exp. Biol.* **213**, 4257–4264 (2010). 21113007 10.1242/jeb.044297 PMC2992466

[44] Hendershot, B. D. & Wolf, E. J. Three-dimensional joint reaction forces and moments at the low back during over-ground walking in persons with unilateral lower-extremity amputation. *Clin. Biomech.* **29**, 235–242 (2014). 10.1016/j.clinbiomech.2013.12.005 24393361

[45] Tranberg, R., Zügner, R. & Kärrholm, J. Improvements in hip- and pelvic motion for patients with osseointegrated trans-femoral prostheses. *Gait Posture*. **33**, 165–168 (2011). 21130654 10.1016/j.gaitpost.2010.11.004

[46] Narres, M. et al. Incidence of lower extremity amputations in the diabetic compared with the non-diabetic population: A systematic review. *PLoS ONE ***12** (2017). 10.1371/journal.pone.0182081 PMC5573217 28846690

[47] Ziegler-Graham, K., MacKenzie, E. J., Ephraim, P. L., Travison, T. G. & Brookmeyer, R. Estimating the prevalence of limb loss in the united States: 2005 to 2050. *Arch. Phys. Med. Rehabil*. **89**, 422–429 (2008). 18295618 10.1016/j.apmr.2007.11.005

[48] Potter, B. K., Rivera, J. A., Anderson, A. B., Souza, J. M. & Forsberg, J. A. What functional outcomes can be expected with osseointegrated prostheses in transfemoral amputations? *Clin. Orthop. Relat. Res. ***483**, 10–1097 (2025). 39898879 10.1097/CORR.0000000000003267 PMC11827995

[49] Rajagopal, A. et al. Full-Body musculoskeletal model for muscle-driven simulation of human gait. *IEEE Trans. Biomed. Eng.* **63**, 2068–2079 (2016). 27392337 10.1109/TBME.2016.2586891 PMC5507211

[50] de Leva, P. Adjustments to Zatsiorsky-Seluyanov’s segment inertia parameters. *J. Biomech.* **29**, 1223–1230 (1996). 8872282 10.1016/0021-9290(95)00178-6

[51] Gottschalk, F. Transfemoral amputation. Biomechanics and surgery. *Clin. Orthop. Relat. Res.* **361**, 15–22 (1999). 10212591

[52] LaPrè, A. K., Price, M. A., Wedge, R. D., Umberger, B. R. & Sup, F. C. Approach for gait analysis in persons with limb loss including residuum and prosthesis socket dynamics. *Int. J. Numer. Method Biomed. Eng.* **34**, (2018). 10.1002/cnm.2936 29111608

[53] Takahashi, K. Z., Kepple, T. M. & Stanhope, S. J. A unified deformable (UD) segment model for quantifying total power of anatomical and prosthetic below-knee structures during stance in gait. *J. Biomech.* **45**, 2662–2667 (2012). 22939292 10.1016/j.jbiomech.2012.08.017

[54] Golyski, P. R. & Sawicki, G. S. Which lower limb joints compensate for destabilizing energy during walking in humans? *J. R Soc. Interface* **19**, (2022). 10.1098/rsif.2022.0024 PMC9156907 35642426

[55] Willems, P. A., Cavagna, G. A. & Heglund, N. C. External, internal and total work in human locomotion. *J. Exp. Biol.* **198**, 379–393 (1995). 7699313 10.1242/jeb.198.2.379

[56] Cavagna, G. A. & Kaneko, M. Mechanical work and efficiency in level walking and running. *J. Physiol.* **268**, 467–481 (1977). 874922 10.1113/jphysiol.1977.sp011866 PMC1283673

[57] West, S. G., Finch, J. F. & Curran, P. J. Structural equation models with nonnormal variables: problems and remedies. In *Structural equation modeling: concepts, issues, and applications*. (ed. *Hoyle R H)* 56–75 (1995).

