# Correction: Comparing the mechanical energetics of walking among individuals with unilateral transfemoral limb loss using socket and osseointegrated prosthetic interfaces

**DOI:** 10.1038/s41598-025-12202-4

**Published:** 2025-08-21

**Authors:** Pawel R. Golyski, Benjamin K. Potter, Jonathan A. Forsberg, Brad D. Hendershot

**Affiliations:** 1 Extremity Trauma and Amputation Center of Excellence, Falls Church, VA USA; 2 Walter Reed National Military Medical Center, Bethesda, MD USA; 3 Uniformed Services University of Health Sciences, Bethesda, MD USA; 4 University of Pennsylvania, Philadelphia, PA USA; 5 Orthopaedics, Department of Surgery, Memorial Sloan Kettering Cancer Center, New York, NY USA

Correction to: *Scientific Reports* 10.1038/s41598-025-93211-1, published online 21 March 2025

The original Article contained errors in Table 1, where the header rows were incorrect. “Socket Interface” and “OI Interface” were inadvertently shifted to the left, and therefore not located above their respective table content. Furthermore, “Time Since Amputation (mo)” was incorrectly placed under “OI Interface”. The correct and incorrect header rows appear below.

Incorrect:

**Table:**
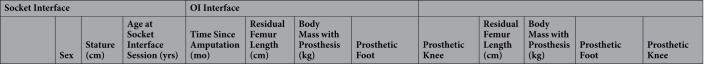

Correct:

**Table:**
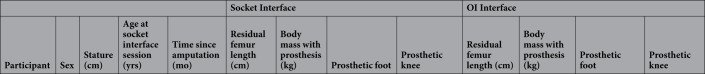

The original Article has been corrected.

