# Supplementary material for: Comparing the mechanical energetics of walking among individuals with unilateral transfemoral limb loss using socket and osseointegrated prosthetic interfaces
Source: Sci Rep. 2025 Mar 21;15:9755. doi: 10.1038/s41598-025-93211-1 (PMC11928544; doi:10.1038/s41598-025-93211-1)
Supplement: Supplementary file 1 — Supplementary Material 1 [file 41598_2025_93211_MOESM1_ESM.pdf]

Title: **Comparing the mechanical energetics of walking among individuals with unilateral transfemoral limb loss using socket and osseointegrated prosthetic interfaces**

Authors: *Pawel R. Golyski[a,b], Benjamin K. Potter[b,c,d], Jonathan A. Forsberg[e], Brad D. Hendershot[a,b,c]

Affiliations:

[a]Extremity Trauma and Amputation Center of Excellence, Falls Church, VA, USA

[b]Walter Reed National Military Medical Center, Bethesda, MD, USA

[c]Uniformed Services University of Health Sciences, Bethesda, MD, USA

[d]University of Pennsylvania, Philadelphia, PA, USA

[e]Orthopaedics, Department of Surgery, Memorial Sloan Kettering Cancer Center, New York, NY, USA

*Corresponding author's email: pawel.golyski.civ@health.mil

Supplementary Figures

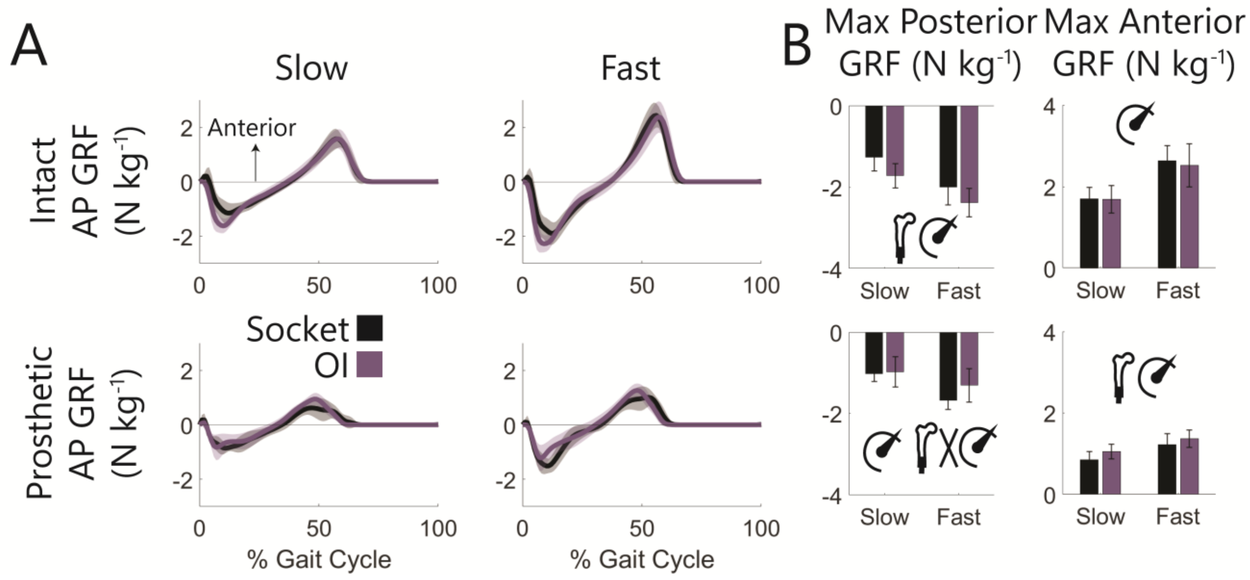

S1 - Across-participant ensemble averaged A) anteroposterior ground reaction forces with (B) associated peak anterior and posterior forces across slow (0.6-1.1 m s^{-1}) and fast (1.1-1.6 m s^{-1}) walking speeds for socket and OI interfaces. Shaded regions and error bars represent ±1 s.d. Values were normalized to each participant's body mass. Significant effects ($P < 0.050$) of interface, speed, and interface-speed interaction were determined using two-way repeated measures ANOVAs and are graphically represented as an osseointegrated femur, speedometer, and their interaction. Statistical results of pairwise comparisons are reported in text.

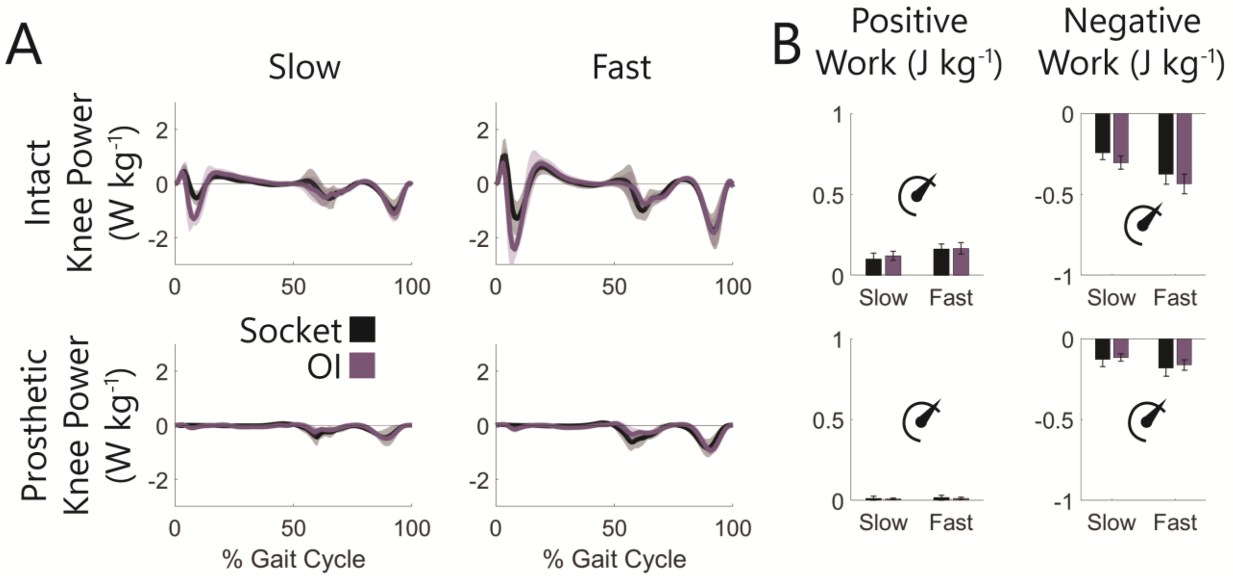

S2 – Across-participant ensemble averaged A) knee powers with (B) associated positive and negative mean work values across slow (0.6-1.1 m s^{-1}) and fast (1.1-1.6 m s^{-1}) walking speeds for socket and OI interfaces. Shaded regions and error bars represent ±1 s.d. Values were normalized to each participant's body mass. Significant effects ($P < 0.050$) of interface, speed, and interface-speed interaction were determined using two-way repeated measures ANOVAs and are graphically represented as an osseointegrated femur, speedometer, and their interaction. Statistical results of pairwise comparisons are reported in text.

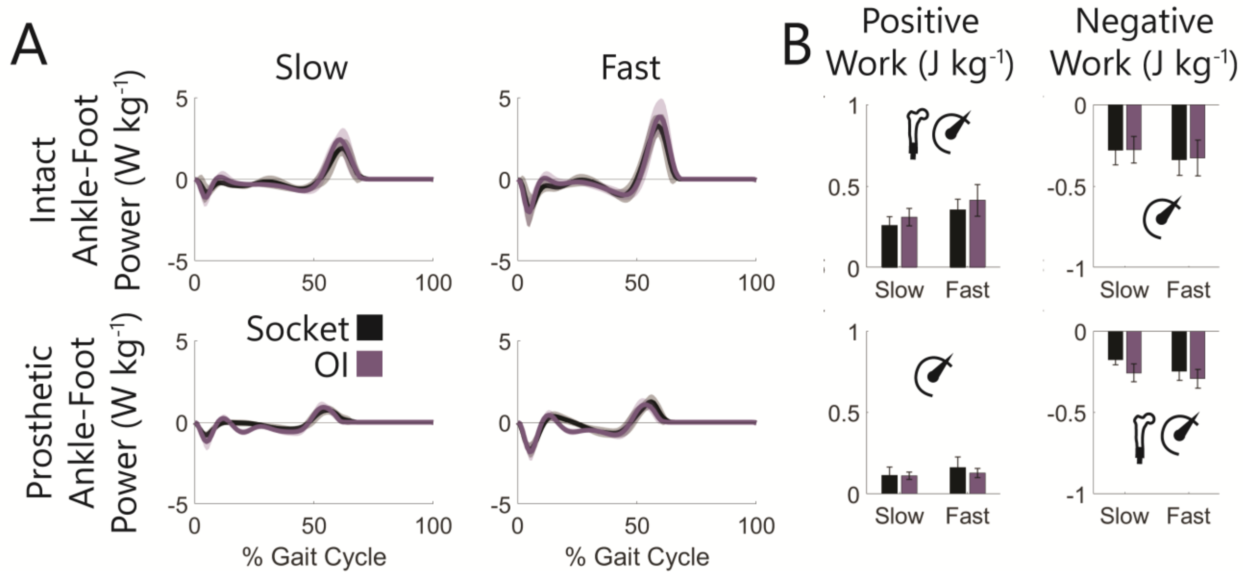

S3 – Across-participant ensemble averaged A) ankle-foot powers with (B) associated positive and negative mean work values across slow (0.6-1.1 m s^{-1}) and fast (1.1-1.6 m s^{-1}) walking speeds for socket and OI interfaces. Shaded regions and error bars represent ±1 s.d. Values were normalized to each participant's body mass. Significant effects (P < 0.050) of interface, speed, and interface-speed interaction were determined using two-way repeated measures ANOVAs and are graphically represented as an osseointegrated femur, speedometer, and their interaction.

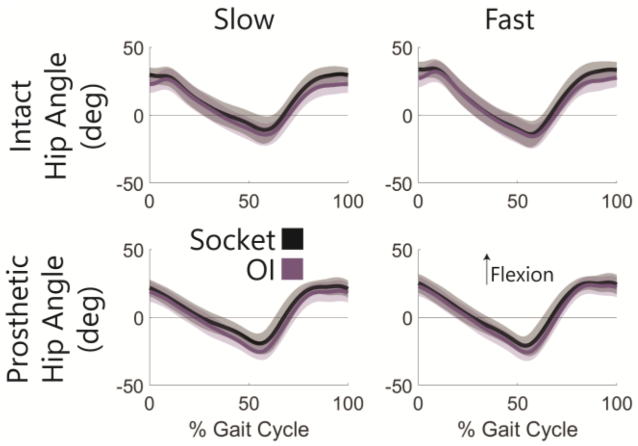

S4 – Across-participant ensemble averaged sagittal hip angles across slow (0.6-1.1 m s^{-1}) and fast (1.1-1.6 m s^{-1}) walking speeds for socket and OI interfaces. Shaded regions represent ±1 s.d.

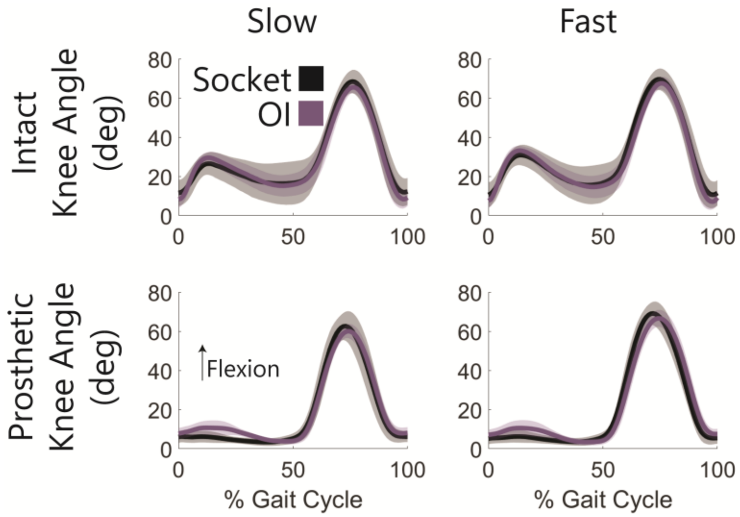

S5 – Across-participant ensemble averaged sagittal knee angles across slow (0.6-1.1 m s^{-1}) and fast (1.1-1.6 m s^{-1}) walking speeds for socket and OI interfaces. Shaded regions represent ±1 s.d.

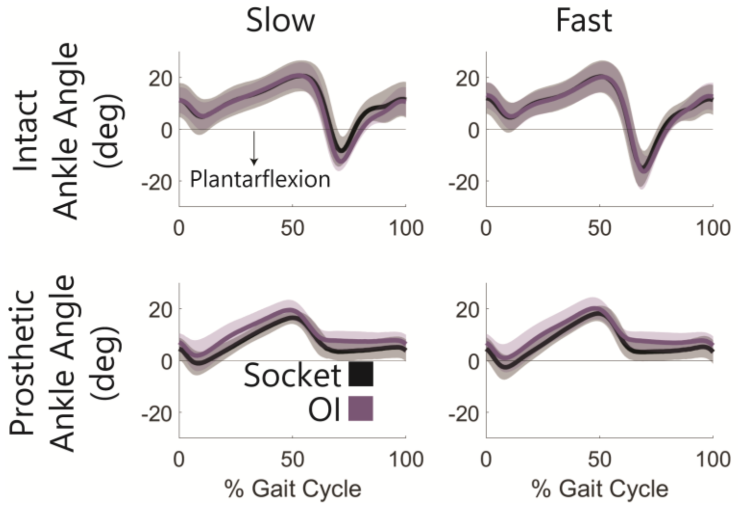

S6 - Across-participant ensemble averaged sagittal ankle angles across slow (0.6-1.1 m s^{-1}) and fast (1.1-1.6 m s^{-1}) walking speeds for socket and OI interfaces. Shaded regions represent ±1 s.d.
